# Supplementary material for: Circadian preferences of birdwatchers in Poland: do “owls” prefer watching night birds, and “larks” prefer daytime ones?
Source: PeerJ. 2020 Mar 2;8:e8673. doi: 10.7717/peerj.8673 (PMC7058107; doi:10.7717/peerj.8673)
Supplement: Supplemental Information 2 [file peerj-08-8673-s002.docx]

List of the journals used in the study to obtain email addresses of Polish ornithologists. Journals arranged in an alphabetical order.

1. *Acta Ornithologica*
2. *Biuletyn Monitoringu Przyrody*
3. *Biuletyn Parków Krajobrazowych Wielkopolski*
4. *Chrońmy Przyrodę Ojczystą*
5. *Dubelt*
6. *International Studies on Sparrows*
7. *Kosmos*
8. *Kulon*
9. *Leśne Prace Badawcze*
10. *Medycyna Weterynaryjna*
11. *Naturalia*
12. *Notatki Ornitologiczne (later: Ornis Polonica)*
13. *Parki Narodowe i Rezerwaty Przyrody*
14. *Polish Journal of Ecology*
15. *Przegląd Przyrodniczy*
16. *Przyroda Śląska Opolskiego*
17. *Ptaki OTOP*
18. *Ptaki Podkarpacia*
19. *Ptaki Polski*
20. *Ptaki Pomorza*
21. *Ptaki Śląska*
22. *Ptaki Wielkopolski*
23. *Roczniki Bieszczadzkie*
24. *Roczniki Naukowe PTOP Salamandra*
25. *Studia i Materiały CEPL w Rogowie*
26. *Sylwan*
27. *Wszechświat*
28. *Zoologica Poloniae*
